# Central Nervous System Metastasis in Epithelial Ovarian Carcinoma: A Case Report and Literature Review

**Published:** 2015-03

**Authors:** Fereshteh Fakour, Hadi Hajizadeh Fallah, Sina Khajeh Jahromi, Parham Porteghali

**Affiliations:** 1 Reproductive Health Research Center, Guilan University of Medical Sciences, Rasht, Iran; 2 Department of Pathology, Guilan University of Medical Sciences, Rasht, Iran

**Keywords:** Ovarian Cancer, Brain Metastasis, Chemotherapy

## Abstract

**Objective:** To report involvement of the central nervous system (CNS) following epithelial ovarian cancer is rare. Advances in management of ovarian cancer by use of primary surgery including abdominal hysterectomy, bilateral salpingo’oophorectomy should attain as complete a cytoreduction as possible and effective platinum-based chemotherapy have prolonged survival.

**Case report:** We present a case involving a 35-year-old Iranian woman diagnosed and treated for primary ovarian cancer in 2002. She underwent optimal cytoreductive surgery and chemotherapy treatment. Eight months after the initiation of therapy, multiple brain metastases without intraperitoneal lesions were found and treated with combination chemotherapy and whole brain radiotherapy (WBRT), without evidence of recurrent disease. The patient died from disease in December 2005.

**Conclusion:** In a patient suffering from neoplasm that rarely metastasizes to CNS, a careful clinical examination and proper therapeutic approach including chemotherapy may lead to prolong survival.

## Introduction

Epithelial ovarian carcinoma (EOC) is fifth most common cancer in women (1). EOC is sdisease that hematogenous metastases are rare at presentation (16%), and remains locoregionally with the most common site of metastatic spread being the pleural cavity (33%), liver (26%) and lung (3%) (2).

Brain metastases are seen rare in ovarian epithelial carcinoma from ovarian cancer are rare. A review of five autopsy studies reported brain metastases in 4% of 712 patients who died with a diagnosis of epithelial ovarian cancer cancer (3). However, the incidence of the central nervous system (CNS) metastasis seems to be increasing (4-7).

Although occasional case report of ovarian cancer with distant metastasis is not associated with intraperitoneal lesions, it is generally recognized that the prognosis of patients with distant metastases is extremely poor (5).

Ovarian cancers disseminate, primarily by several ways. The first one continuity the second one Lymphatic dissemination to pelvic and para-aortic lymph nodes (40% of patients at stage III-IV disease) as well as the third one to the peritoneum is common. At the time of diagnosis, bone or brain metastases are rarely present and that is not related to the histology or grading of the tumor (8).

Surgery, irradiation, and chemotherapy constitute the treatment regime for prolonging survival, although the prognosis for these patients have remained poor (4).

The median survival time after the diagnosis of brain metastases was 6 months and 27 days (6).

In this study, we reported a rare case of ovarian cancer with brain metastases that was not associated with intraperitoneal lesion, occasionally reported.

## Case report

**Figure 1:**
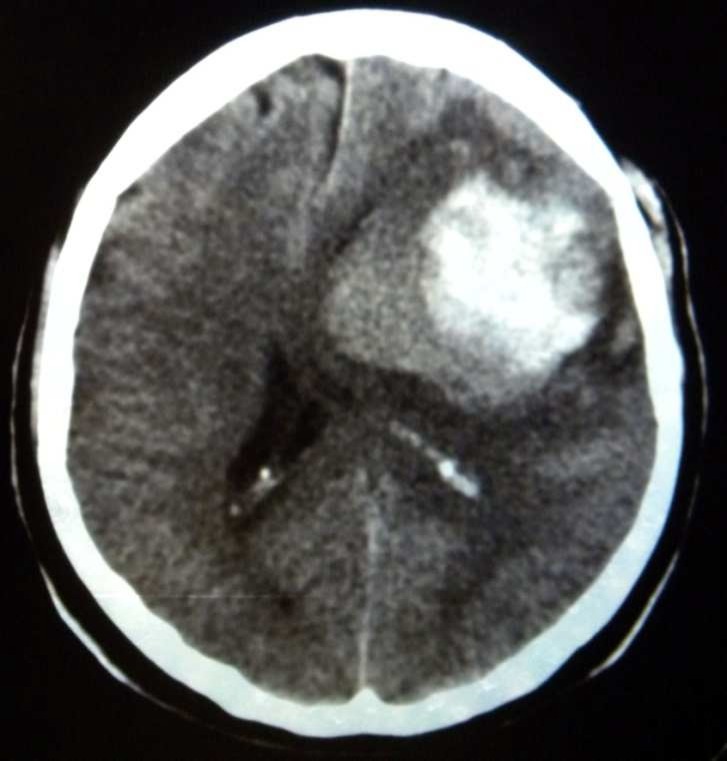
Metastatic brain lesion with irregular border

**Figure 2:**
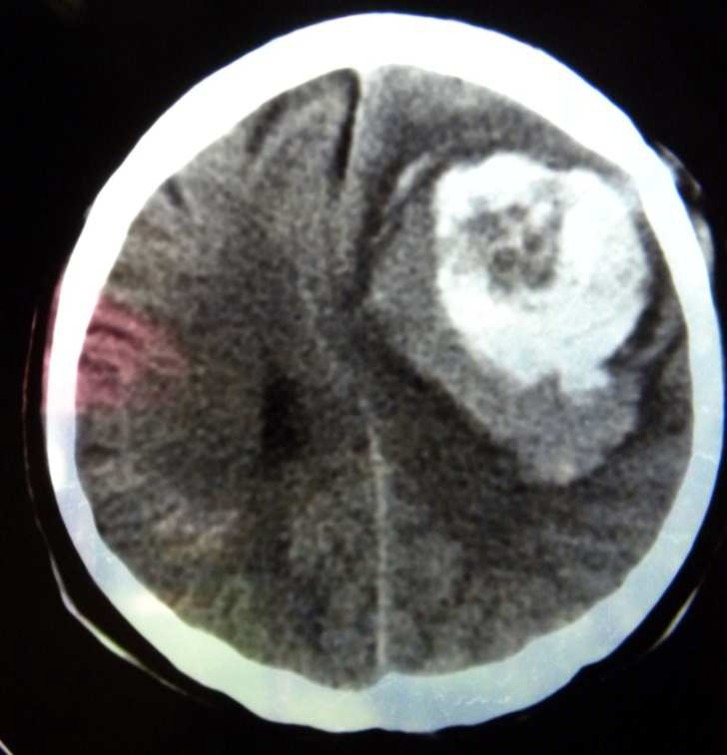
Metastatic brain lesion with central necrosis

A 35-year-old Iranian woman (GII, PII) initially presented with abdominal distention in May 2002. Imaging studies confirmed an ovarian tumor with a large volume of ascites. Cytodiagnosis of ascites revealed adenocarcinoma. Based upon these finding along with a clinical diagnosis of ovarian cancer, patient underwent laparotomy. The operation consisted of abdominal hysterectomy, bilateral adenexectomy, and omentectomy. The post-surgical pathological studies revealed serous cyst adenocarcinoma of ovary (stage IIIC). Thereafter, she received 6 courses ofcombination chemotherapy consisting ofTaxole (175 mg) and carboplatin (AUC 6) and patient showeda complete response to chemotherapy. The result of post treatmentabdominal pelvic computed tomography (CT) scan and serum CA125 levels were normal. The patient was under close observation and had been well for 8 months, without any symptom and sign of recurrence. Eight months after therapy, the patient presented suddenly withtonic- clonic seizures. The result of brain CT scan revealedmetastatic brain lesion (Figures 1 and 2), whereasabdominal pelvic CT scan findings and serum CA125 levels were normal. Abdominal and rectovaginal examinations did not detect any ascites or mass. The patient received whole brain irradiation (WBRT; 3500CGY) as well asa combination chemotherapy consisting of gemcitabine and carboplatin that subsequently decreased theneurological complains and she had no problem during 18 months ofpost-treatment follow-up. In December 2005, she developed visual disturbance and gait abnormality. Brain CT showed multiple brain metastases. The patient’s general health condition deteriorated and she died after a month.

## Discussion

We reported the status of an ovarian cancer patient who developed metastatic brain disease a few months after diagnosis. Brain metastases following ovarian cancer are uncommon, although reports have suggested that the prevalence rate may be increasing (4-7).

In 1966, Bergman reported autopsy findings on 86 patients who died of epithelial ovarian carcinoma; only one patient (1.2%) had brain metastases (7).

Systemic metastases from ovarian carcinoma are frequent, but they seldom affect the brain. In our case study, the patient was treated for ovarian cancer with surgery and chemotherapy. After the end of chemotherapy, the patient developed cerebral metastases from ovarian carcinoma and underwent whole brain radiation and chemotherapy. Brain metastasis can occur during or after adjuvant chemotherapy; however, the best treatment strategies are not well known (9).

The most common type of epithelial ovarian cancer reported in the literature is the serous adenocarcinoma (10, 11). As in our case with moderated differentiated serous adenocarcinoma, the International Federation of Gynecology and Obstetrics (FIGO) stage IIIc has been correlated with increased incidence of brain metastasis. The time between the primary tumor diagnosis and cerebral involvement has been 5 times shorter in stage 3 or 4 disease that in stage 1 or 2 (11).

Two hypotheses can be postulated to explain the apparent increase in CNS involvement among patients suffering from other types of cancers, including leukemia, lymphomas, prostate, and soft tissue sarcomas. Firstly, the use of effective combination chemotherapy, especially containing cisplatin for ovarian cancer, gives patients the possibility of long-term survival, permitting occult CNS metastases to become overt. Secondly, the CNS may be a "pharmacologic sanctuary" that is not always readily reachable by the drugs even with high intravenous dosage (7).

In a study by Cohen et al, 35% of patient had single and 65% had multiple brain metastases. An analysis of possible factors predicting prognosis, including ovarian tumor FIGO stage, grade and histological type, showed none of predictive factors for developing single versus multiple brain metastases (6). A study reported presence of multiple brain metastases to be adversely associated with survival (11). But another study showed no difference in survival between single versus multiple brain metastases in ovarian carcinoma (6).

The median time after diagnosis and CNS metastases were 14.5 months (12). In our patient, CNS metastases developed after she received platinum-based chemotherapy, 8 months after diagnosis. She had no intraperitoneal metastases at the time of brain metastases and that is considered a rare event.

The median overall survival time of patients after the diagnosis of brain metastasis was 6.27 months, and survival probabilities of patients at 6 months, 1 year and 5 years were 54%, 31% and 5%, respectively (6).

Clinical manifestation includes motor weakness, seizure, headache, confusion, and speech disturbance (5, 7, 11). In our patient, seizure developed at the time of diagnosis without any neurological deficits.

For patients with multiple brain metastases, WBRT with or without chemotherapy remain the treatment of choice. Poor penetration of the blood-brain barrier is considered to be a limiting factor for routine use of systemic chemotherapy. Recent studies with systemic chemotherapy have shown objective responses with improved survival for patients with CNS metastases among patients with primary breast cancer and germ cell tumor. Objective responses and survival benefit have also been documented even in patients with primary EOC (13).

Many of the chemotherapy (e.g. carboplatin) are unable to permeate the blood-brain barrier, and thus cannot protect the patients from development of brain metastases (5). In our case, chemotherapy showed no effect in development of CNS manifestation (it developed 2 months after the end of chemotherapy).

Metastatic brain disease originating from a primary ovarian carcinoma is treated with surgery, irradiation and chemotherapy (12). It is generally believed that there are limited indications for surgery in patient with multiple brain metastases. However, previous experience at M.D. Anderson Cancer Center of University of Texas has demonstrated that surgical removal of all lesions in selected patients with multiple brain metastases increased survival time, with an outcome similar to that of patients undergoing surgery for single metastasis (14). In our patient with multiple brain metastases, after radiotherapy and chemotherapy, she had a normal brain CT scan and showed no clinical brain manifestation after treatment. Brain metastases in our patient were diagnosed early due to tonic- clonic seizures. However, early diagnosis by brain CT scanning during developing any symptoms (headache, fatigue, and gait ataxia) followed by multimodality treatment (surgery, radiotherapy, and chemotherapy) may improve overall survival in these patients.
